# GWideCodeML: A Python Package for Testing Evolutionary Hypotheses at the Genome-Wide Level

**DOI:** 10.1534/g3.120.401874

**Published:** 2020-10-22

**Authors:** Laura G. Macías, Eladio Barrio, Christina Toft

**Affiliations:** * Departament de Genètica, Universitat de València, Burjassot, Valencia, Spain; † Departamento de Biotecnología de los Alimentos, Instituto de Agroquímica y Tecnología de los Alimentos (IATA), CSIC, Paterna, Valencia, Spain; ‡ Program for Systems Biology of Molecular Interactions and Regulation, Institute for Integrative Systems Biology (I2SysBio; UV-CSIC), Valencia, Spain

**Keywords:** Positive selection, Comparative genomics, Genome analysis, Molecular evolution, Protein sequence analysis, Python

## Abstract

One of the most widely used programs for detecting positive selection, at the molecular level, is the program *codeml*, which is implemented in the Phylogenetic Analysis by Maximum Likelihood (PAML) package. However, it has a limitation when it comes to genome-wide studies, as it runs on a gene-by-gene basis. Furthermore, the size of such studies will depend on the number of orthologous genes the genomes have income and these are often restricted to only account for instances where a one-to-one relationship is observed between the genomes. In this work, we present GWideCodeML, a Python package, which runs a genome-wide *codeml* with the option of parallelization. To maximize the number of analyzed genes, the package allows for a variable number of taxa in the alignments and will automatically prune the topology to fit each of them, before running *codeml*.

With the rise of the genomic era, comparative analyses are gaining interest and are becoming more feasible due to the increase of available genomes. Testing evolutionary hypotheses to measure selective pressure in coding sequences is a common approach in evolutionary biology projects. To do this, there are different bioinformatic tools and resources like the PAML package (Yang 2007)⁠. Within this package, *codeml* allows estimating the ratio (ω) between non-synonymous and synonymous substitutions in protein-coding sequences. The assumption that synonymous mutations accumulate neutrally, implies that ω can be used as a measure of the selective pressure on the coding sequence. In a neutral evolutionary scenario, this ratio will remain equal to one while it will be less or greater than one under purifying and positive selection, respectively. *Codeml* can be run with different assumptions, including the most used: ω is constant along the whole coding sequence but it can vary among branches (branch model), ω remains constant among branches but it can differ among codon sites in a coding sequence (site model) or assuming that *ω* varies both among branches and sites (branch-site model). The desired model, along with other parameters, is given to *codeml* through the control file, along with the coding-sequence alignment of orthologous genes and the species tree.

Implementing *codeml* in an automatic workflow for a genome-wide approach has its challenges. Among these is the negative correlation between the number of genomes and the number of orthologs shared among them implying that increasing the number of genomes to improve the statistical power of the method will result in reducing the number of analyzed genes. However, the lack of one particular gene, in any of the analyzed species, might not be critical for the analysis if the number of remaining orthologs is substantial.

In this work, we provide a Python package, GwideCodeML, that can be used for running *codeml* on a set of orthologous coding sequences under site, branch or branch-site models. This package automatically generates the files necessary to run the *codeml* program including pruning of the topology to match each of the alignments in cases where some taxa are missing compared to the species tree. A dataset of two *Saccharomyces* species is used for testing our program. The package allowed the use of several outgroup species without decreasing the number of genes included in the analysis.

## Methods

### Input files

**Figure 1:**
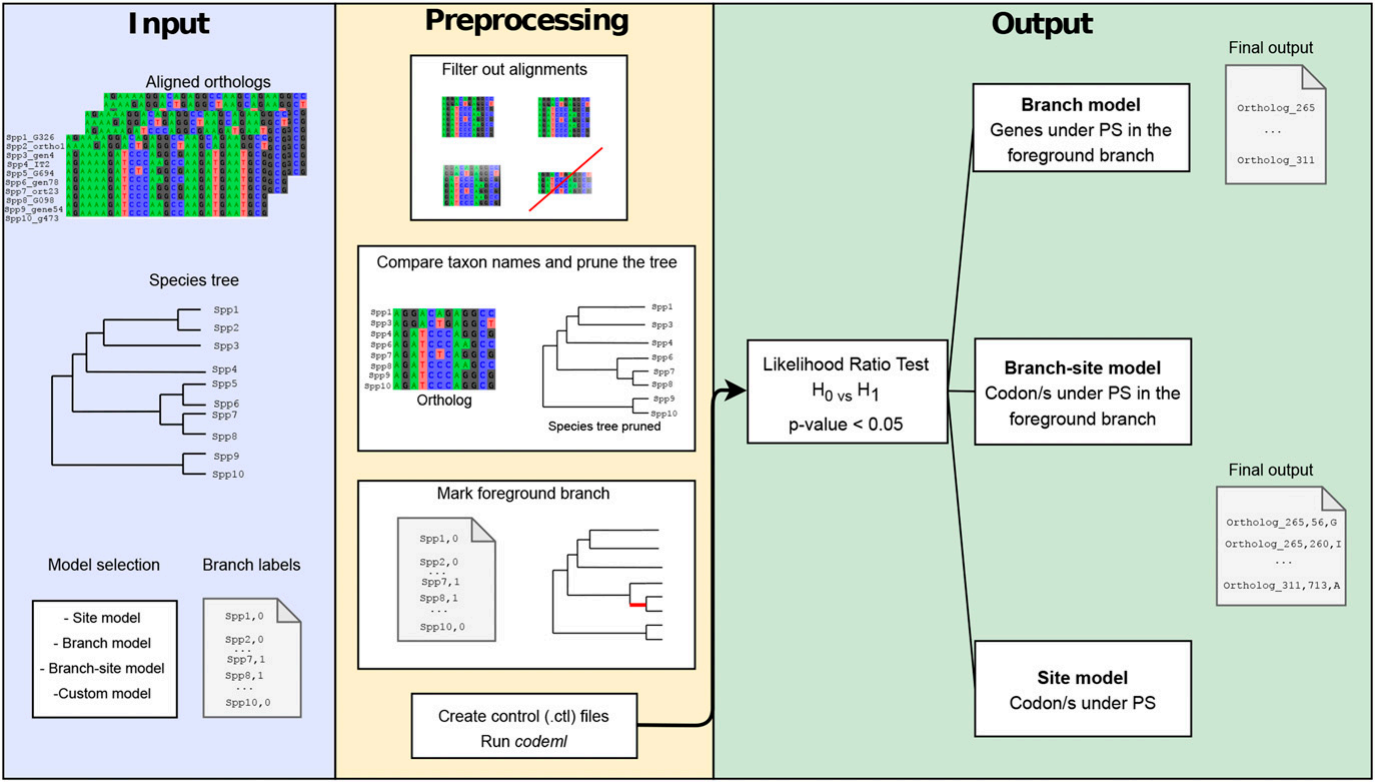
Schematization of the GwideCodeML workflow divided in the three main parts: required input, preprocessing steps necessary for creating control *codeml* files necessary for testing both null and alternative hypotheses. The last part of the pipeline shows the output file obtained after running LRTs on the *codeml* results file. This output file contains all the candidate genes of being under positive selection.

GWideCodeML requires as input, a directory with codon-aligned orthologous sequences in FASTA format and a NEWICK-format tree topology containing all the species included in the analysis (Figure 1). A common denominator between the names within the FASTA file and the taxa (*e.g.*, species and/or strain names) within the tree, is also required. It should be noted that the pipeline itself does not handle possible duplication events and the users should make sure the FASTA files contain a maximum of one sequence per species/strain. Besides, the users need to provide optional configuration parameters such as the model to be tested. Furthermore, it is possible to set a minimum number of species/strains, either belonging to the foreground branch and/or to the outgroup, to filter out alignments with low statistical power. Whether the users decide to test a branch, branch-site model, or to set a threshold, a text file with a list of branch label information is also required as input. In the case of branch and branch-site model, an integer should be added after the branch label to indicate which clade the taxon belongs to. Specifying integers other than 0 and 1 - used for the background and foreground, respectively - will allow for multiple branch testing. In these cases, the workflow will be run for each of the specified foreground clades (integers greater than 0).

### Pipeline execution

Codon-aligned sequences are passed through the workflow if: i) only one gene per taxon is found and ii) they pass the threshold of the number of sequences, which by default is set to 0. Hereafter, sequence names are compared between the alignment file and the species tree. The tree will be pruned until it only contains the taxa present within the alignment.

From this step onwards, the workflow performance will depend on the model selected by the user (branch, site, branch-site, or custom). In the case of the branch or branch-site model, the new tree created (pruned or original) will be used to indicate the foreground branch. Hereafter, the control files necessary to run both the alternative and null hypotheses are created and *codeml* (included in the PAML v4.9 package) is run with each of them. This is the most time-consuming step, however, the package allows parallelization of this task when the user provides a maximum number of threads to be used by the program set by the *-p* optional parameter. Once *codeml* has finished, GWideCodeML parses the output files to perform a Likelihood Ratio Test (LRT) to assess the level of significance between the two hypotheses. This last part is optional, allowing the users to analyze *codeml* results by themselves or letting the pipeline do it for them. When branch or branch-site models have been selected, GWideCodeML will optionally (-gene_trees option) run FastTree (Price *et al.* 2010) on each of the alignments and compare the topologies of the gene and species tree focusing on the foreground branches. In cases where the studied clade is not monophyletic between the two trees, the user will be informed.

### Nested models implemented in the pipeline

Three nested models have been implemented in GWideCodeML for their execution. The branch model allows dN/dS ratio to vary among branches assuming this ratio remains constant among codons (Yang 1998; Yang and Nielsen 1998)⁠. This model is useful for detecting positive selection acting on a particular branch and it uses M0 (one-ratio model) and two-ratios model (Goldman and Yang 1994) as null and alternative hypothesis testing, respectively.

Site model assumes dN/dS ratio might be different among codons of the coding sequence provided (Nielsen and Yang 1998; Yang 2000)⁠. To test this, model M1a or NearlyNeutral and M2a or PositiveSelection (Yang and Nielsen 1998; Yang *et al.* 2005) are built-in on the workflow as null and alternative hypotheses, respectively. Finally, the branch-site model is a combination of the two previous approaches as it allows dN/dS ratio to vary among branches and codon sites. Model A null and model A have been used in our package to test both null and alternative hypotheses (Yang *et al.* 2005; Zhang *et al.* 2005)⁠.

The three described models are nested and therefore a LRT can be applied to assess the level of significance of the null hypothesis. In cases where the *p*-value obtained from the LRT is lower than 0.05, the null hypothesis is rejected and this gene is further investigated. Additionally, it is possible to vary the level of significance by applying the Bonferroni’s test correction (Miller 1981) when multiple branches are tested. This method has been proved to be effective and usable (Anisimova and Yang 2007) in the correction of the level of significance for null hypothesis rejection depending on the number of hypotheses tested.

Rejecting the null hypothesis means that the alternative hypothesis is more likely than null for the given species topology and alignment. In addition to the LRT, it is necessary to check whether dN/dS ratio is greater than one in the foreground branch (branch or branch-site models) to determine if a gene is a candidate gene of being under positive selection. Moreover, in the cases of site and branch-site models, *codeml* offers two methods for calculating posterior probabilities for site classes to identify codons under positive selection when the LRT is significant. These methods are naïve empirical Bayes (NEB) and Bayes empirical Bayes (BEB) (Yang *et al.* 2005)⁠. PAML authors highly recommend ignoring NEB output and use BEB instead so the package extracts codon positions with a probability greater than 0.9 of being under positive selection according to BEB results when the LRT is significant.

### Additional options and modules

GWideCodeml contains a number of additional scripts and options which can aid the users in preparing the input files or acquire further information about the data. They include i) a module which allows the users to align their codon sequences with three different aligners (Mafft, Muscle or Prank). ii) another module which helps the users to create the alignment necessary to reconstruct a robust species phylogeny by selecting the genes containing sequences for all species included in the analysis. iii) The workflow works with the provided species phylogeny, however, it is possible to set the parameter -gene_tree, and the program will flag the genes where the studied clade is not monophyletic in the species and gene trees. iv) In the case of the branch and branch-site models, the option -dnds will parse the foreground and background ω’s into a separate output file. v) a module which performs a Bonferroni’s test correction when multiple branch testing has been performed.

### Output files

The final output of the pipeline is a text file with a list containing genes under positive selection according to the analysis performed. Additionally, when the site or branch-site model is selected, gene names are accompanied with the codon positions in the alignment under positive selection. A gene may have one or more codon positions under positive selection.

### Case-study

A genome dataset of two species of *Saccharomyces*, *S. cerevisiae* and *S. kudriavzevii*, was used for testing the performance of our package. These genomes had already been analyzed in a previous work (Macías *et al.* 2019)⁠, where the dataset was conformed of nine genomes: four annotated genomes of each of the *Saccharomyces* species and *Torulaspora delbrueckii* as the outgroup species. For testing this package we increased this dataset, particularly the outgroup, with 18 annotated yeast genomes from The Yeast Gene Order Browser (Byrne and Wolfe 2005)⁠, a database which also contains information on the homology of different species of the Saccharomycotina subphylum.

In the analysis of selection performed in Macías *et al.*, (2019) with nine genomes, the number of genes analyzed was limited by the number of common orthologous genes shared among them: 4165 genes. In the tested dataset here, which included 26 yeast genomes, only 2753 genes were shared among all species. By setting a minimum number of three background species necessary for testing the evolutionary hypotheses on a gene and four strains in the foreground branch, our pipeline almost doubled the genes analyzed to 4920 genes.

### GWideCodeML compared to other software

Several bioinformatics resources have been developed to simplify and/or automate positive selection analyses. Some of these tools are aimed at facilitating the running of *codeml* and visualize the results in one task, meaning that the users need to run the program gene by gene (Stern *et al.* 2007; Delport *et al.* 2010; Steinway *et al.* 2010; Xu and Yang 2013)⁠. There are also other tools created for running *codeml* in a genome-wide framework like GwideCodeML. Most of these tools have overlapping features among them and when compared to the package presented here (Table 1). The main benefit of our package, when compared to the others, is the combination of features that makes it possible to test site, branch and branch-site models on filtered orthologs with a variable number of sequences among them. PosiGene (Sahm *et al.* 2017) is the only one of the three published programs which is able to run with a variable number of taxa in the orthologs, it does this by generating a gene tree for each run or as GWideCodeML, prunes the provided species tree to fit each of the alignments. When looking for positive selection, it is important to have a correct topology, however, both species and gene trees have their problems. In the case of gene trees, incorrect topology can occur due to long-branch attraction and natural variation between genes due to the stochastic nature of mutations (Castresana 2007; Jeffroy *et al.* 2006; Rokas and Carroll 2006). On the other hand, occurrences of horizontal gene transfer will result in the species topology being incorrect. GWideCodeML uses the species tree, but the package will flag the genes where the conservation of the taxa in the studied clade is not the same in the species and gene topology. Furthermore, PosiGene⁠, along with POTION (Hongo *et al.* 2015)⁠, can perform some of the pre-processing steps, although they have been developed to run only one specific model. In contrast, LMAP (Maldonado *et al.* 2016)⁠ is the most flexible regarding the models it offers, the three different nested models along with custom settings, although it lacks the pre-processing steps necessary to run them on a dataset composed of orthologs with a heterogeneous number of sequences. Other features which set GWideCodeML apart from the other three software is that it allows for testing multiple branches in the same run and it contains a module for multiple hypothesis testing.

**Table 1:** Overlapping features between GWideCodeML and other bioinformatics tools. *^1^ Built-in models: site model (SM), branch model (BM), branch-site model (BSM). *^2^ PosiGene generates a new tree for each gene, where GWideCodeML prunes the provided species tree

Feature	LMAP	POTION	PosiGene	GWideCodeML
Built-in models *^1^	SM, BM, BSM	SM	BSM	SM, BM, BSM
Run costume models	Yes	—	—	Yes
Easy branch labeling	Yes	—	Yes	Yes
Automatic pruning *^2^	—	—	Yes	Yes
Filter out low quality orthologs	—	Yes	Yes	Yes
Multithreading	Yes	Yes	Yes	Yes

### Data availability

The GWideCodeML package is implemented in Python and is freely available at https://github.com/lauguma/gwidecodeml

## Results And Discussion

This package not only allowed us to analyze a higher number of genes but it has also dramatically increased the statistical power of our study by including more species. If we compared the number of significant genes obtained using only nine genomes and the branch-site model tested here, we can see our package also increased the number of positive results (Table 2). More specifically, we obtained 30 and 32 genes under positive selection in *S. kudriavzevii* and *S. cerevisiae* branches, respectively, in Macías *et al.*, (2019). However, using GWideCodeML under the branch-site model assumption, we obtained 137 and 96 in *S. kudriavzevii* and *S. cerevisiae* branches, as gene candidates to be under positive selection. Increasing the number of outgroup species has been demonstrated to affect the power of detecting sites under positive selection (Goodswen *et al.* 2018)⁠.

**Table 2:** Case-study results. Number of detected genes under positive selection after running GwideCodeML twice, one for each branch, using the three built-in nested models. *^1^. In site models, there is no dN/dS ratio variation among branches, therefore, it was run once

Model	Nested models (null *vs.* alternative hypotheses)	No. genes under positive selection in *Sk* branch	No. genes under positive selection in *Sc* branch
Branch	M0 *vs.* two-ratios	83	31
Branch-site	MA_null_ *vs.* MA	137	96
Site	M1a *vs.* M2a	32^*1^	32^*1^

Besides, our package facilitates the running of additional models such as branch and site models. The combination of the results of the three approaches tested can provide more depth on how positive selection has been acting on the studied clade.
